# Polysaccharides Are Effective Inhibitors of Natural Gas Hydrate Formation

**DOI:** 10.3390/polym15071789

**Published:** 2023-04-04

**Authors:** Alsu Venerovna Fakhreeva, Vasily Viktorovich Nosov, Alexander Iosifovich Voloshin, Vladimir Anatolyevich Dokichev

**Affiliations:** 1 Ufa Institute of Chemistry, Ufa Federal Research Center, Russian Academy of Sciences, 450054 Ufa, Russia; 2 RN–BashNIPIneft LLC, 450103 Ufa, Russia

**Keywords:** gas hydrate, gas hydrate formation, polysaccharides, “green” inhibitor of gas hydrate formation

## Abstract

This review covers the types and applications of chemical inhibitors of gas hydrate formation in the oil and gas industry. The main directions of the development of new types of highly effective and environmentally safe “green” kinetic hydrate inhibitors (KHIs) based on biopolymers are analyzed. The structure, physicochemical properties, efficiency of gas hydrate formation inhibition, and commercial prospects of polysaccharides in preventing and controlling the formation of gas hydrates are considered. The criteria for their selection, current experimental data, and the mechanism of inhibition are presented. Recent research in the development of cost-effective, efficient, and biodegradable KHIs for industrial applications in the oil and gas industry is also presented.

## 1. Introduction

The formation of technogenic gas hydrate deposits occurs in downhole equipment (casing and tubing) and surface communications (oil collection system, separation, and stabilization units). It is one of the factors that complicate the development and operation of oil and gas fields during drilling operations and oil production. Hydrates clog the reservoir pores, thereby reducing their permeability and depositing on the walls of wells and oil field equipment, and they reduce the throughput capacity of production wells and process pipelines, leading to losses in oil production and additional costs resulting from a decrease in the flow rate and the need to periodically stop wells for anti-hydration measures [1,2,3,4,5,6,7,8,9,10]. The formation and agglomeration of gas hydrates are favored by the presence of free water and certain thermodynamic conditions (temperature, pressure, composition of the gas and liquid phases); as a result, hydrate plugs are formed, leading to a decrease in the throughput capacity of wells and pipelines [1,2,3]. Inhibitors are used to prevent hydrate formation, and current trends in the field of ecology suggest the use of biodegradable reagents, the use of which will reduce the anthropogenic load on the environment during oil production. As such reagents, biopolymers are the most prospective, among which polysaccharides occupy a priority position. Natural gas hydrates are associated not only with complications in the processes of oil production, but also with the fact that they can become an almost inexhaustible source of fossil hydrocarbons. Colossal deposits of methane hydrate are concentrated in the bowels of the earth in permafrost conditions [11] and in the depths of the World Ocean, spread all over the world and accessible to most countries [12,13]. Hydrocarbon reserves (mainly methane) are estimated at 2 × 10^16^ m^3^ [14].

Gas hydrates are clathrate compounds formed by the interaction of water and low-molecular-weight gases, such as hydrocarbons, C_1_-C_4_, CO_2_, H_2_S, and N_2_, under certain thermobaric conditions [15]. Hydrates of natural gases are solid compounds in which water molecules bound together by hydrogen bonds are cells containing a molecule or molecules of petroleum gas (methane, ethane, propane, butane, CO_2_, H_2_S, N_2_) [16,17]. The unit cell sizes of the gas hydrate crystal framework are 1–2 nm, and the size of a statistically significant phase formation (at least 1000 unit cells) is in the range of 15–20 nm. The structure of a clathrate compound is determined by the guest molecule, and in this regard, several types of elementary gas structures are distinguished: cubic I, cubic II, and hexagonal III. The kinetics of the formation, dissociation, and agglomeration, as well as the morphology, thermodynamics, and properties, of gas hydrates, including water–gas and oil–gas media, are presented in well-known works [15,16,17,18,19,20,21,22,23,24,25,26,27,28,29,30,31,32,33,34,35,36,37].

The rate of gas hydrate formation, decomposition, and agglomeration depends on a driving force of the oil–gas flow, pressure, temperature, water phase state, water mineralization, and gas and oil compositions [17,38,39,40].

**Figure 1:**
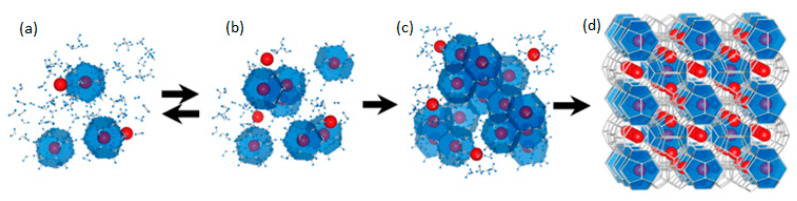
Labile cluster model of hydrate nucleation: (**a**) labile clusters, (**b**) agglomeration of clusters, (**c**) primary nucleolus, and (**d**) hydrate crystal. Reprinted from Ref. [32].

**Figure 2:**
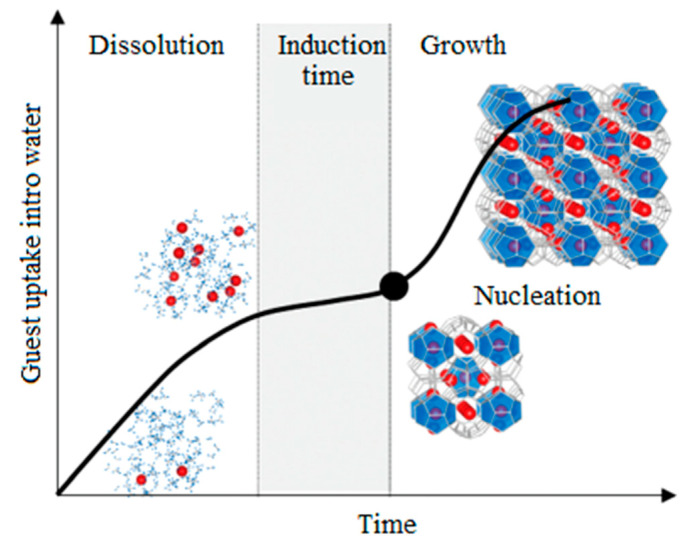
Typical time dependence of the hydrate crystallization process. Reprinted from Ref. [32].

Hydrate crystallization occurs in two main stages (Figure 1). The first stage is known as nucleation, which is the appearance of a crystalline formation from disordered or liquid molecules. The second stage is crystal growth, followed by an increase in particle size, leading to the formation of three thermodynamically stable canonical hydrate crystal structures (sI, sII, and sH). A typical time dependence of the hydrate crystallization process is shown in Figure 2 [32].

Nucleation and growth of gas hydrate crystals can occur:

- At the water (ice)–gas interface;
- In the volume of free gas saturated with water vapor;
- In the volume of gas-saturated water;
- In the volume of the gas-saturated oil fluid.

When considering the ways of nucleation of hydrates, homogeneous and heterogeneous are distinguished. Nucleation is the initial stage of the process of forming a new structure through self-organization. With homogeneous nucleation, the nucleation of the hydrate phase arises directly from the initial phase and is usually observed in systems without any impurities and is considered stochastic, i.e., critical nucleation is formed due to local thermodynamic fluctuations of the system, and the resulting clusters of molecules can either increase or contract as a result of thermodynamic fluctuations [41]. Near the equilibrium curve, the probability of the formation of homogeneous nuclei is small and can take a very long time.

In practical conditions, the formation of hydrates by homogeneous the nucleus formation is very unlikely and proceeds by heterogeneous formation of nucleus. The results of a number of studies show the important role of the presence of liquid–vapor, liquid–surface, liquid–vapor–surface phase separation and the processes of surface adsorption of guest molecules, which increases the probability of the hydrate nucleus formation [17,41]. The time interval between the establishment of supersaturation and the formation of critical nuclei is called the induction time. Independent studies have shown that supersaturation does not always guarantee the formation of hydrates (Figure 2) [17,32,41,42]. The induction time is determined by the metastability of the solution, given the fact that the nucleation process is stochastic; a large spread for experiments is explained by the presence of many uncontrolled factors, the influence of which can be leveled by the conditions of the experiment. The following mechanisms are currently considered have been proposed to explain hydrate nucleation: Classical Nucleation Theory, Labile Cluster Hypothesis, Nucleation at Interface Hypothesis, Blob Formation Mechanism [17,41]. A number of kinetic models of gas hydrate growth have been proposed. The kinetic models were based on chemical processes taking into account heat and mass transfer, changes in concentration or fugacity [43].

## 2. Formation of Gas Hydrates in Oil and Gas Production Processes

**Figure 3:**
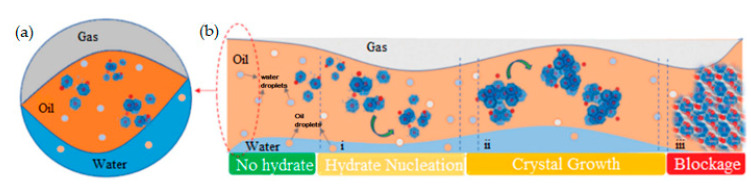
Schematic model of a multiphase flow in a pipeline, where hydrate crystal aggregates can flow in gaseous, oleic, and aqueous phases and/or deposit onto the pipe solid surface; (**b**) schematic diagram of the drivers of hydrate crystal formation and aggregation in hydrocarbon transportation pipelines and three-step process of (**i**) hydrate nucleation, (**ii**) crystal growth, and (**iii**) blockage. The transverse (**a**) and longitudinal (**b**) cross-section of the pipeline. Reproduced from Ref. [32].

The formation of technogenic gas hydrate deposits in a water–oil–gas dispersed medium is mainly a three-stage process: (i) hydrate nucleation; (ii) hydrate crystal growth and agglomeration; (iii) formation of a hydrate plug and blocking of the flow line, which can lead to a complete blockage of the processing pipeline (Figure 3).

**Figure 4:**
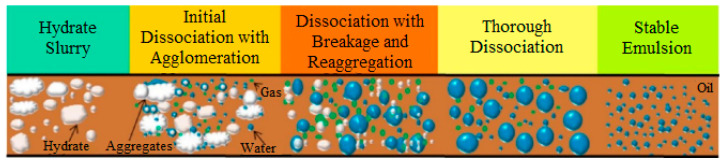
Conceptual model of the hydrate decomposition process in an oil-dominated flow system. Reproduced with permission from Ref. [37] Copyright 2018 Elsevier.

Dissociation of gas hydrates can occur in multiphase systems during the flow of hydrates in a water–oil–gas dispersed medium, because hydrate decomposition is influenced by the collision effect, including its own kinetic velocity, the effect of mass and heat transfer, and the nature of the multiphase flow of the oil and gas fluid. The kinetics of hydrate dissociation with respect to heat and mass transfer inside pipelines is important for the hydrate core/plug breakup, and it should be taken into account in predicting complications in oil pipeline transport (Figure 4) [37].

**Figure 5:**
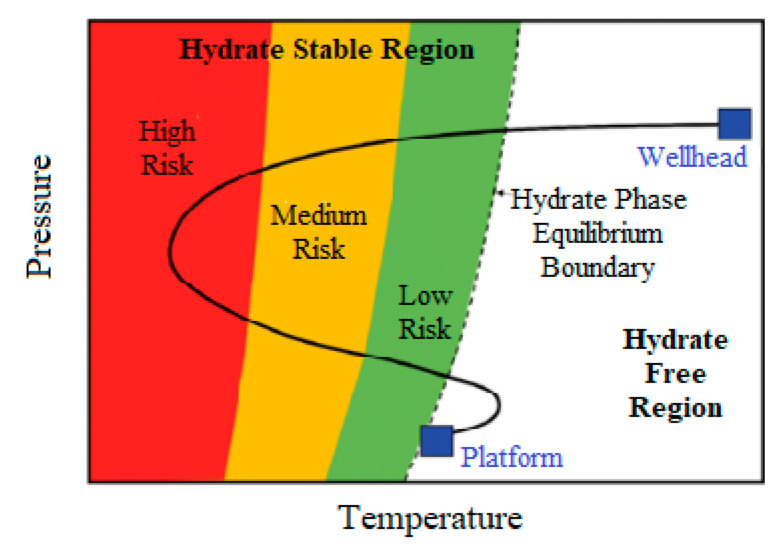
Illustration of a risk map for hydrate formation in oil and gas production Reproduced with permission from Ref. [36] Copyright 2017 Springer Nature.

In [36], the concept of developing hydrate formation risk maps is presented, taking into account areas with a low, medium, and high probability, and determining the areas of the production system in which hydrate formation is likely (Figure 5). Such maps make it possible to predict hydrate formation in production equipment using pressure and temperature, and to implement certain technological solutions to minimize or eliminate risks.

## 3. Prevention and Control of Gas Hydrate Formation in Oil and Gas Production Processes

Chemical, technological, and physical methods are used to prevent the formation of gas hydrates in downhole equipment (inter-tube space, tubing) and ground communications (oil collection system, separation, and stabilization units) [10,44,45,46,47,48]. Currently, the most widely used chemical methods are based on the use of gas hydrate formation inhibitors, whose action is based on changing the conditions of formation, inhibiting the nucleation and growth of gas hydrates, and preventing the formation of large agglomerations [2,6,9,47,48,49,50,51,52,53,54,55,56,57,58,59,60,61,62,63]. The use of hydrate formation inhibitors is a convenient and economical technology [48], the efficiency and reliability of which have been demonstrated for many years in industrial conditions [62,63,64,65,66,67].

According to the mechanism of action, gas hydrate formation inhibitors are divided into three types [48] (Table 1):

- Thermodynamic hydrate inhibitors, whose action is based on the shift the hydrate-liquid-vapor equilibrium of gas hydrate formation towards lower temperatures and high pressures (methanol, ethanol, ethylene glycol, glycols, salt solutions, etc.);
- Kinetic hydrate inhibitors (KHIs), which are water-soluble polymers that prevent or delay the nucleation and/or growth of hydrates (homo- and copolymers of N-vinylcaprolactam, N-isopropylacrylamide, and N-vinylpyrrolidone);
- Anti-agglomerates (AAs), which are surfactants that do not stop nucleation but stop the agglomeration (sticking together) of gas hydrate crystals.

Methanol, ethylene glycol, diethylene glycol, and aqueous solutions of electrolytes (sodium and potassium chlorides) are typical thermodynamic inhibitors of gas hydrate formation. The use of these reagents is not environmentally safe and leads to an increase in the cost of oil and gas production and transportation; they are mostly used in high dosages (10–50%) [44,48].

Currently, preference is given to kinetic inhibitors of hydrate formation (KHIs) [48,52,57,58,59,60,68,69,70,71], which are effective in significantly lower concentrations (0.1–3.0%) over traditional thermodynamic inhibitors. A great advantage of kinetic-type inhibitors is the dosage, which is many times lower than the dosage of thermodynamic inhibitors. This significantly reduces capital and operating costs.

Kinetic inhibitors reduce the rates of nucleation and crystal growth of gas hydrates, allowing the multiphase flow to pass through a section of the pipeline that is dangerous for gas hydrate formation. The effectiveness of their use is evaluated by the degree of subcooling and the time of induction [72]. Known KHIs have the ability to prevent hydrate formation at temperatures around 12–15 °C, and the best inhibitor to date has inhibited hydrate formation under 24.1 °C hypothermia conditions [73]. However, the use of kinetic inhibitors of hydrate formation is limited by the low subcooling temperature and water content (40–60%) of the water–oil fluid, since the hydrate suspension under these conditions becomes too viscous and the inhibitor efficiency is low [70].

**Figure 6:**
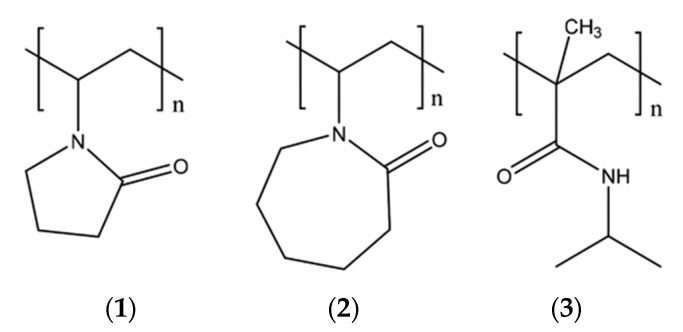
Kinetic inhibitors of gas hydrate formation: poly-N-vinylpyrrolidone (PVP) (**1**), poly-N-vinylcaprolactam (PVCap) (**2**), poly-N-isopropyl methacrylamide (PNIPMAm) (**3**).

The main components of most modern commercial KHIs are one or more water-soluble amide-containing copolymers or polymers containing hydrophobic and hydrophilic fragments, for example, poly(N-vinylpyrrolidone) (PVP), poly(N-vinylcaprolactam) (PVCap), and poly(N-isopropylacrylamide) (PNIPAM) [48,57,74]. In [74], the comparative effectiveness of a number of commercial and well-known KHIs (Luvicap 21 W (copolymer of N-vinylpyrrolidone:N-vinylcaprolactam 2:1; MW 21,000 g/mol; BASF), Luvicap 55 W (N-vinylpyrrolidone:N-vinylcaprolactam copolymer 1:1; 2000−4000 BASF), PVP (polyvinylpyrrolidone; K15 9000 Ashland Chemical Co., Singapore, Singapore), PNIPAM (poly N-isopropylacrylamide 8500), and PAM (polyacrylamide; 5,000,000 Sigma-Aldrich, St. Louis, MO, USA)) and the following activity were detected: Luvicap 21 W ≥ Luvicap 55 W > PNIPAM ≥ PVP > PAM (Figure 6).

Anti-agglomerates are used in small doses of 0.1–3.0% of the volume of the aqueous phase, which makes it possible to achieve high technical and economic indicators as a result of a significant reduction in the dosage and an increase in the effectiveness of the inhibitory effect [48,57,75,76,77]. Currently, quaternary ammonium-based surfactants are commercially available as AAs. Anti-agglomerates are sorbed on the surface of gas hydrates and prevent their agglomeration and the formation of traffic jams in multiphase gas–water–oil flows, thus ensuring multiphase transport of hydrocarbons in the gas hydrate formation mode without the deposition of hydrates in field communications.

Singh and Suri considered the formation of hydrate nuclei and their inhibition using kinetic acrylamide-based hydrate inhibitors [54]. In addition, the effects of the KHI concentration, polymer molecular weight on hydrate inhibition, and various kinetic methods were presented. The induction time and subcooling were the estimated kinetic parameters. It is established that the constant cooling method is the most convenient method for checking the KHI operability, in which the induction time decreases with increasing cooling rate. Moreover, polymers with larger or more hydrophobic functional groups show better inhibition of nucleation, and with an increase in the polymer concentration, the induction time in this case increases linearly. Furthermore, it is concluded that if KHIs have an alkyl group attached to their main chain (methylated polymers), they perform better than non-alkylated polymers. Consequently, N-isopropylmethacrylamide-co-methacrylamidopropyltrimethylammonium chloride (NIPMAM-MAPTAC) stands out as a very highly effective KHI among all studied acrylamide-based KHIs, with a 1000 times higher nucleation time. However, Liu et al. found that as the size of the lactam ring increases in the KHI structure, its ability to inhibit nucleation increases [49]. Ke and Chen discussed the nature of the resulting natural gas hydrates, their microscopic characteristics, synergists of inhibitors, and various mechanisms of inhibition [69].

In the oil and gas industry, water-soluble polymers such as polyvinylpyrrolidone (PVP), PVCap, and Gaffix VC-713 are mainly used as kinetic inhibitors to control hydrate formation. Although the vast majority of traditional KHI polyamide polymers have low levels of acute toxicity and bioaccumulation, few commercial KHIs show good biodegradability, and for this reason, there is always some concern about the long-term chronic toxicity of KHI metabolites in the environment [69]. The high cost of cleaning a barrel of water is also another disadvantage of using traditional KHIs. Although KHIs are not as toxic, there is still a requirement from many corporate and government authorities to achieve much higher levels of biodegradability, and this has limited the use of KHIs in some oil- and gas-producing regions. Due to the requirement of high biodegradability, efficiency, and compatibility with the borehole fluid of AI in the field of oil and gas production and transportation, kinetic inhibitors of a new generation have recently been developed, which are characterized by an increased rate of biodegradation and do not have a negative impact on the environment [51,53,78,79,80,81,82,83,84,85,86,87,88,89].

In some fields, the degree of subcooling is too high for KHIs, so they are used together with traditional thermodynamic inhibitors (usually low-molecular-weight polar compounds) to reduce subcooling to the point where KHIs can be used [89,90]. Compositions have been developed that prevent the formation of gas hydrates, which include kinetic-type inhibitors and thermodynamic-type inhibitors. Kinetic inhibitors reduce the rate of hydrate formation, while thermodynamic inhibitors lower the temperature of their formation compared to non-inhibited systems. The combined use of both inhibitors leads to a synergistic effect and reduces hydrate formation to a greater extent. It was shown [90] that ethylene glycol is a good synergist for KHIs, and 1% of the inhibitor can be replaced with ethylene glycol without reducing the inhibitory properties. It is possible to use other oil field reagents that exhibit synergistic properties with other chemicals. KHIs can be used together with corrosion inhibitors, quaternary ammonium salts, and alcohol and glycol solvents [57,69,81,91,92,93,94,95,96,97,98].

Various research groups have developed and tested a large number of new non-amide compounds on all types of gas hydrates, including KHIs based on amines, amine oxides, phosphonates, sulfonates, acrylates, urethanes, etc. [51]. Some of the non-amide KHIs with suitable hydrophobic groups exhibit better inhibitory properties and a higher turbidity temperature in salt solutions than some of the well-known commercial KHIs containing an amide group, which indicates great potential for further development in this direction. The advantages and disadvantages of the main categories of non-amide polymer KHIs are presented in [51].

## 4. Polysaccharides Are Promising “Green” Inhibitors of Gas Hydrate Formation

**Figure 7:**
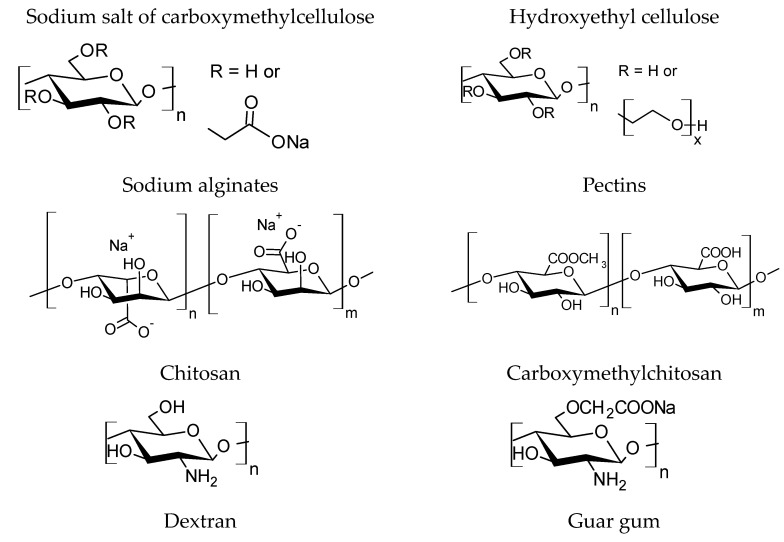

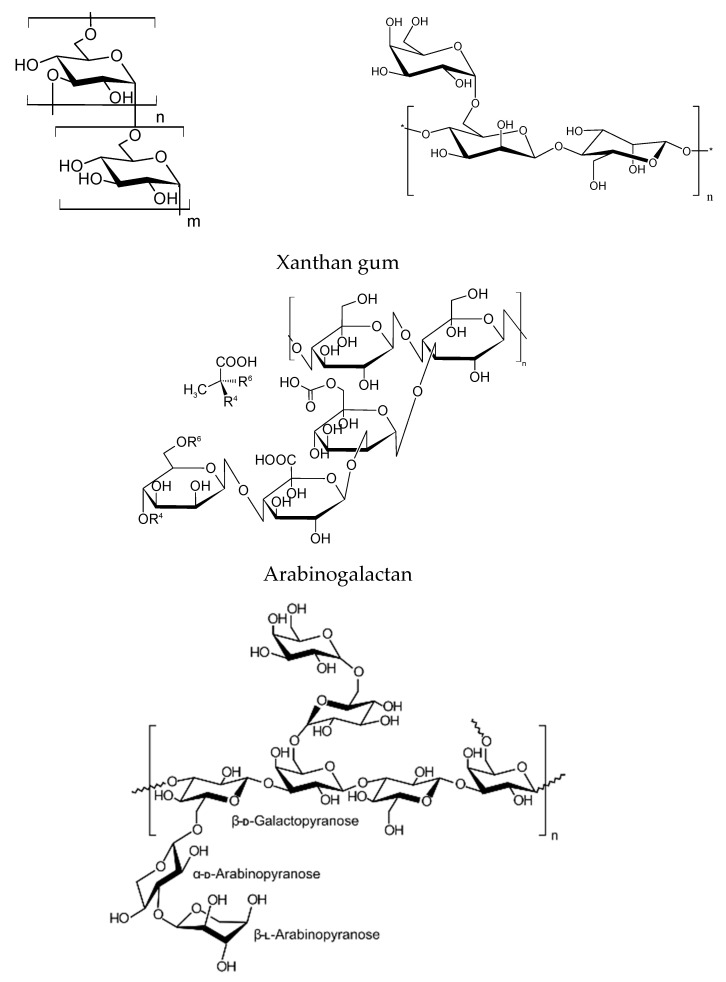
Structural formulas of polysaccharides.

Polysaccharides are polymer molecules consisting of long linear or branched chains of monosaccharide units (glucose, mannose, galactose, rhamnose, etc.) connected by glycosidic bonds (Figure 7). The number of repeating monosaccharide units in the main polymer chain ranges from 40 to 3000. Similar to proteins and glycosaminoglycans, which are also common natural polymers, polysaccharides can be extracted from a variety of sources, such as plants, microorganisms, and animals. They are biodegradable, non-toxic, and considered low-risk for the environment [99,100,101,102,103,104,105]. Polysaccharides are included in the OSPAR list [106] and accord with the provisions of OSPAR. Their use and discharge into the water in areas of the North-East Atlantic, including the North Sea, are permitted.

Polysaccharides such as cellulose, starch, guar, xanthan gum, guar gum, gellan gum, velan gum, and their derivatives meet the requirements of strict environmental regulations, are highly effective, and are used in the oil and gas industry for drilling oil and gas wells, for hydraulic fracturing, in oil recovery enhancement processes, and as sediment formation inhibitors [107,108,109].

A considerable amount of experimental data on various aspects of hydrate formation inhibitors based on polysaccharides and their derivatives have been published in the literature, including in several reviews covering specific aspects of hydrate formation and inhibition [32,43,49,50,51]. Kelland M.A. studied many commercially available natural polysaccharides [51] and showed the potential for creating new “green” inhibitors of low-concentration gas hydrate formation based on them. The history of polysaccharide LDHI development was reviewed by Kelland M.A. et al. [57]. The obtained data destroyed the earlier assumption that the presence of hydrophobic groups in the structure determines the high efficiency of amide KHIs, and for this reason, natural polysaccharides are not of interest. It was also noted that aqueous solutions of natural polysaccharides are characterized by high viscosity and gelation, which will lead to complications when pumping these solutions. It has been shown that the reason for the poor thermodynamic affinity of natural guar polysaccharides (M = 1.6 × 10^6^) and xanthanum (M = 2.2 × 10^6^) is the strong decrease in the non-combinatorial entropy during dissolution, which manifests itself in positive values of the Flory–Huggins entropy parameter, lying in the range of 0.8–0.9, which is consistent with the strong aggregation observed in polysaccharide solutions [110].

However, experimental data [79,81] have shown that polysaccharides, as a result of the formation of hydrogen bonds of glucose-containing compounds, are formed in the form of galactose, galactopyranose fragments, and carboxyl groups with water molecules in the oil and gas fluid and in the structure of gas hydrates in the stage of nucleation. When they reach a critical size, they can inhibit gas hydrate formation and exhibit both thermodynamic and kinetic inhibitor properties.

Wang et al. studied the structural properties and efficiency of various biopolymers in inhibiting gas hydrates [79]. Five polysaccharides, namely, gum arabic, sodium alginate, guar gum, carboxymethylchitosan, and starch, were investigated as potential inhibitors of natural gas hydrate formation, and their characteristics were compared with commercial KHIs such as PVP K90. All polysaccharides increased the maximum subcooling of hydrate formation and inhibited hydrate growth well below their corresponding maximum subcooling. Guar gum showed the best characteristics, most likely due to the presence of anhydroglucose side groups in its structure. 

The ratio of the integrated Raman peak intensities for the large (*I_L_*) and small (*I_S_*) hydrate cages decreased in the presence of polysaccharides, i.e. a smaller number of large hydrate cage were occupied by methane. For guar gum, the *I_L_*/*I_S_* ratio was the lowest, most likely due to the presence of anhydroglucose side groups in its structure.

**Figure 8:**
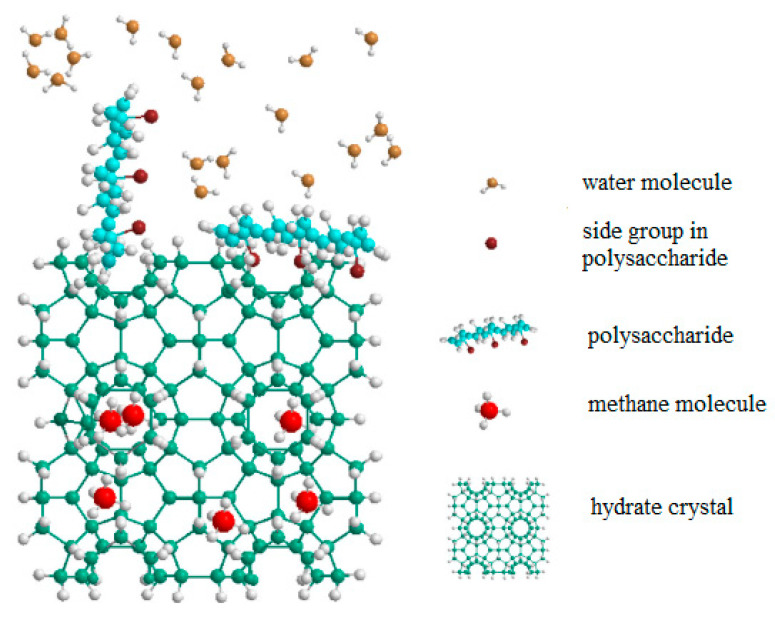
Schematic of the possible hydrate growth inhibition mechanisms of polysaccharides Reproduced with permission from Ref. [79] Copyright 2019 Elsevier.

The properties of these polysaccharides can be explained by the binding of the anhydroglucose unit of polysaccharides to the open cavities of the hydrate structure. Hydroxyl groups in the anhydroglucose link contribute to a strong interaction between the polymer and water molecules, thereby preventing gas molecules from entering the hydrate structure, which leads to further inhibition of hydrate crystal growth, and a decrease in the gas content and its absorption. The difference in the effectiveness of polysaccharide inhibition is most likely due to their different side chains. The anhydroglucose group in the guar gum skeleton structure can increase its adsorption on the hydrate surface, thereby leading to the lowest *I*_L_/*I*_S_ ratio compared to other polysaccharides. The multi-branched complex molecular structure of gum arabic may be the reason for it having the worst inhibition rates among the polymers tested. A possible mechanism of hydration inhibition by polysaccharides has been suggested (Figure 8) [79].

A study of guar gum with different molecular weights showed that guar gum with a higher molecular weight showed better kinetic inhibition (KHI) of methane hydrate formation [78]. Guar gum with a molecular weight of 1.7 × 10^6^ g/mol showed an average induction time of 643 min under subcooling of 10–11 °C. In the study of Wang et al. guar gum (Mn = 5.38 × 10^5^ g/mol) was reported to produce an average maximum subcooling of 8.5 °C on methane hydrates, which is better than that of commercial PVPC90 [79]. In addition, San Atgar and Peivandi reported that commercial guar gum provided a longer service life and induction time than PVP on methane hydrates at different concentrations [84]. Other gums, such as gum arabic and xanthan gum, also showed some kinetic inhibitory effect on methane hydrates, but they did not provide better results than guar gum [78,79,84,111]. Xanthan gum has a synergistic effect on commercial KHIs [112,113].

Pectin is a type of polysaccharide; the main ingredient is a polymer of D-galacturonic acid [99,100,101]. Pectins have been studied as KHIs by various research groups [59,114,115,116,117,118]. Xu et al. reported that pectin extracted from pomelo peel showed excellent methane hydrate inhibition characteristics at a concentration of 0.25 wt% compared to PVCap (molecular weight = 1410 g/mol) [115]. Studies using molecular dynamics modeling show that inhibition of pectin action may be related to the interaction between pectin molecules and water molecules through hydrogen bonds [116]. Experimental results showed that the number of carboxyl groups affects the overall polarity (i.e., low-methoxylated pectin) and significantly improves the effectiveness of hydrate inhibition. Compared to PVCap, highly methoxylated pectin showed a comparable trend and slightly better performance at most concentrations used. The authors, however, noted a noticeable difference in the mechanism of hydrate formation. The use of low-methoxylated pectin in an optimal concentration can provide inhibition efficiency up to three times higher than that of PVCap under high-subcooling conditions [118].

Operational tests of kinetic inhibitors of hydrate formation showed that all pectins were capable of delaying the onset of hydrate nucleation, while highly methoxylated pectin was the most effective in delaying nucleation at a concentration of 0.5 wt%. For further studies of pectin as a kinetic inhibitor of hydrates, chemical modification (substitution, hydrolysis, amidation, etc.) of the molecular structure and functional properties of pectin is of great interest. It is possible to obtain pectin derivatives with varying hydrophobicity and hydrophilicity.

The cellulose polymer is a linear polysaccharide whose chain consists of D-glucose units linked together via β (1,4)-glycosidic bonds [99,100,101]. Yakub et al. studied the efficiency of kinetic inhibition of commercial Na-CMC using carbon dioxide and methane and found that Na-CMC has a mild kinetic inhibitory effect on methane hydrates [111,114]. Na-CMC at a concentration of 0.1–0.5 wt% is the optimal thickening reagent for systems of drilling fluids, with an inhibitory effect for the safety of drilling with the possibility of the formation of gas-hydrate-containing deposits [119].

Lee and co-workers reviewed a number of commercially available cationic starches as KHIs [120]. They found that their kinetic inhibitory effect on hydrocarbon hydrates is determined by the polymer structure [111,114]. The results of the Talaghat study [121,122] showed that oxidized starch offered better kinetic inhibition characteristics than commercial PVP (MM = 10,000 g/mol) when using methane, propane, isobutane, and carbon dioxide as hydrate-forming gases. In addition, the efficiency of kinetic inhibition by oxidized starch can be enhanced by polyethylene oxide and polypropylene oxide [122]. It is interesting to note that potato starch showed a promoting effect on methane hydrate formation, rather than inhibition [123].

In developing new “green” inhibitors of gas hydrate formation, Voloshin A.I. et al. [62,63,124,125,126] studied the comparative ability of a number of available biodegradable polysaccharide polymers, sodium, and ethanolammonium salts of carboxymethylcellulose, dextran, and arabinogalactan to inhibit gas hydrate formation under the conditions of a quasi-equilibrium thermodynamic experiment under isothermal conditions (24.5 °C). Carboxymethylcellulose (Na-CMC) is a chemical modification product of linear cellulose, and dextran is a 3,6-branched natural polysaccharide. The natural water-soluble polysaccharide arabinogalactan (AR) is a highly branched polysaccharide with a main chain composed primarily of 1-3-linked β-D-galactopyranose residues, most of which carry side branches at C-6. Arabinogalactan side chains contain β-D-galactopyranose residues and their 6-O-(C) and 3,6-di-O-glycosylated fragments, as well as unsubstituted and 3-O-substituted β-L-arabinofuranose residues together with β-D-arabinofuranosyl, β-L-arabinopyranosyl, and some other fragments [127].

Gas hydrate formation and the effect of polysaccharides were studied in a high-pressure cell in a polythermal mode. A mixture of hydrocarbon gases typical of the composition of petroleum gas was used as a model gas-hydrate-forming medium [125]. The inhibitory activity was determined in comparison with the widely used thermodynamic anti-hydrate reagent, methanol.

The effectiveness of the studied compounds as inhibitors of gas hydrate formation was evaluated according to a number of criteria, determined by Equations (1) and (2).

The first criterion allowed for evaluating the thermodynamic properties of the polysaccharides. The pressure difference (∆*P*_0_,_ing_) at the beginning of gas hydrate formation is determined by the absence (*P*_0_) and presence of a polysaccharide (*P*_0_,_ing_) at specified concentrations under isothermal conditions:

∆*P*_0,ing_ = *P*_0_,_ing_ − *P*_0_; (1)

For the selected gas mixture, *P*_0_ = 143 bar at *t*_0_ = 24.5 °C.

The second criterion allowed for estimating the change in the rate of gas hydrate formation in the presence of a polysaccharide. It was assumed that the change in pressure in the cell from time Δ*P*_ing_ = *P*_0,ing_ − *P*_ing_ is proportional to the amount of hydrate formed *N*_i_, the maximum pressure change Δ*P*_ing,max_ = *P*_0_,_ing_ − *P*_ing,min_). The formal kinetic equation in this case can be represented as an equation for a first-order reaction:

(2) $$ln\frac{\Delta P_{ing,max}}{\Delta P_{ing,max}-\Delta P_{ing}}=rt$$

where *P*_0,ing_ is the pressure at the beginning of gas hydrate formation in the presence of a polysaccharide, bar; *P*_ing_ and *P*_ing,min_ are the hydrate formation pressure in the presence of a polysaccharide at time *t* and the minimum pressure in the cell, bar; *r*—effective rate constant of gas hydrate formation, c^−1^_._

**Figure 9:**
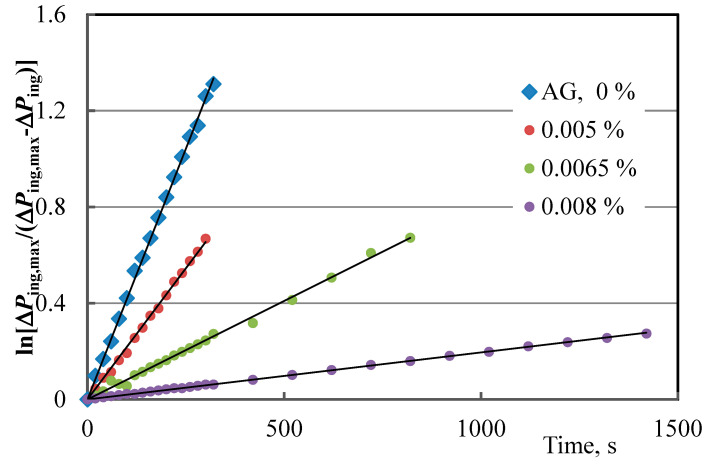
Kinetics of hydrate formation in the presence of AG at *t* = 24.5 °C.

**Figure 10:**
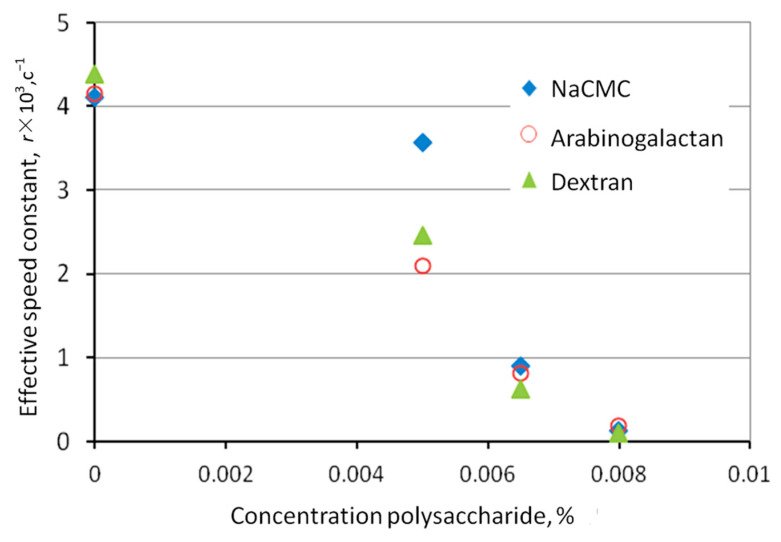
Dependence of hydrate formation rate constants in the presence of polysaccharides.

The gas hydrate formation of the model gas mixture in the presence of 0.005, 0.0065, and 0.008% polysaccharides demonstrated high inhibitory activity (Table 2). Without an inhibitor, the gas hydrate formation process began at 143 bar, whereas in the presence of polysaccharides, the formation of gas hydrates occurred in the region of higher pressures: 155–185 bar. Dextran, Na-CMC, and arabinogalactan as thermodynamic inhibitors are 170–270 times more effective than methanol. Dextran is superior in terms of inhibition efficiency, reduction in the gas hydrate formation rate, and prolonged induction time, when compared to Na-CMC and arabinogalactan. Since the pressure drop of gas hydrate formation increases with an increase in the concentration of polysaccharides and the rate of gas hydrate formation decreases, the mechanism of action of the studied polysaccharides can be attributed to both thermodynamic and kinetic inhibitors (Figure 9 and Figure 10, Table 2).

It is known that the molecular weight of water-soluble polymers has a significant effect on their inhibitory properties. In this regard, the activity of three types of carboxymethylcellulose sodium salts with substitution degrees of 0.7 and 0.9, differing in molecular weights, was studied. In the series of studied samples, namely, Na-CMC-90, Na-CMC-250, and Na-CMC-700, the highest inhibitory activity, which was 400 times more effective than that of methanol, was demonstrated by a polysaccharide with a molecular weight of 250,000 Da at a concentration of 0.05% (Table 3). The sodium salt of carboxymethylcellulose with a mass of 700,000 Da had no effect on the formation of hydrates. This is probably due to the fact that in an aqueous solution, the molecules of this polysaccharide are in the form of a “tangle”, and as a result, interaction with gas hydrates practically does not occur [92].

It is assumed that polysaccharides apparently interact with gas hydrate clusters or their nuclei, adsorbing on the surface of gas hydrate particles due to hydrogen bonds and, thus, preventing their further growth, which manifests itself in a decrease in the rate of hydrate formation. An increase in the induction period of hydrate formation [78,79] indicates that the polysaccharide, regardless of the nature of the functional groups, exhibits the properties of KHIs.

**Figure 11:**
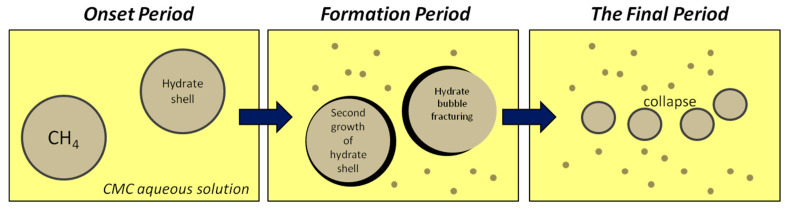
Scheme of the decomposition of the hydrate shell of a gas hydrate in the presence of Na-CMC.

It is assumed that the inhibition of gas hydrate formation under the action of Na-CMC (Figure 11) occurs as a result of the electrostatic interaction of ionized carboxyl groups and sodium cations with water molecules in the hydrate shell, and in general for polysaccharides, as a result of the formation of hydrogen bonds with oxygen atoms and OH groups of D-glucose fragments [81,128].

Of particular interest are nitrogen-containing cellulose derivatives contained in the side chain, along with hydroxyl and carboxyl groups and ethanolammonium ions, which can interact with gas hydrates and are of interest as complex oil field reagents, not only for gas hydrate formation inhibition but also salt deposition and corrosion.

**Figure 12:**
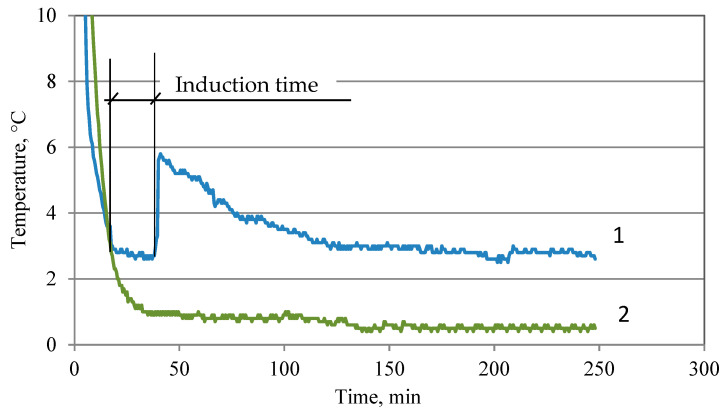
Effect of monoethanolammonium salt of carboxymethylcellulose on hydrate formation in THF-H_2_O. 1—temperature change in the absence of polysaccharides; 2—temperature change in the presence of 0.1% polysaccharides.

**Figure 13:**
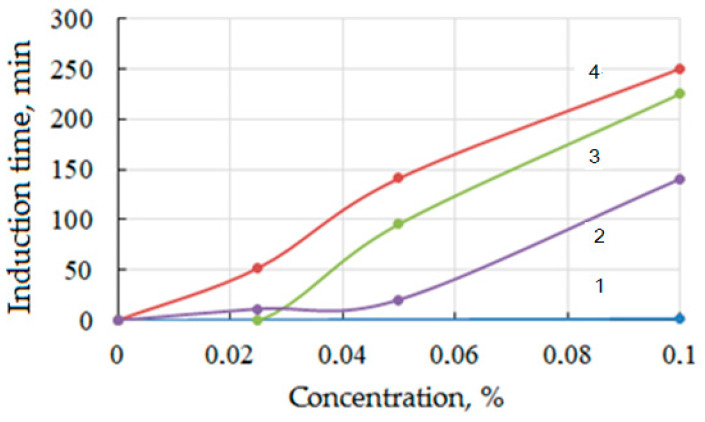
Dependence of induction time of hydrate formation on methanol concentration (1) and mono- (2), di- (3), and triethanolammonium salts of carboxymethylcellulose (4).

Among ethanolammonium salts, the monoethanolammonium salt of carboxymethylcellulose shows the greatest effectiveness in inhibiting the formation of tetrahydrofuran (THF) hydrates. When its concentration increases from 0.02 to 0.1%, the induction time required for the nucleation and subsequent growth of crystals increases by a factor of five (Figure 12 and Figure 13). At a concentration of 0.1% salt, hydrate is practically not observed during the entire experiment (5 h). When switching to the di- and triethanolammonium salts of CMC, the efficiency of inhibition decreases (Figure 13). At a concentration of 0.1% polysaccharides, the induction period of hydrate formation with mono-, di-, and triethanolammonium groups increases by 350, 225, and 150 times.

The studied ethanolammonium salts of carboxymethylcellulose apparently interact with gas hydrate clusters and their nuclei, adsorbing on the surface of gas hydrate particles and forming hydrogen bonds not only with carboxymethylcellulose, but also with ammonium groups, thus preventing their further growth and the formation of gas hydrates of a critical size.

**Figure 14:**
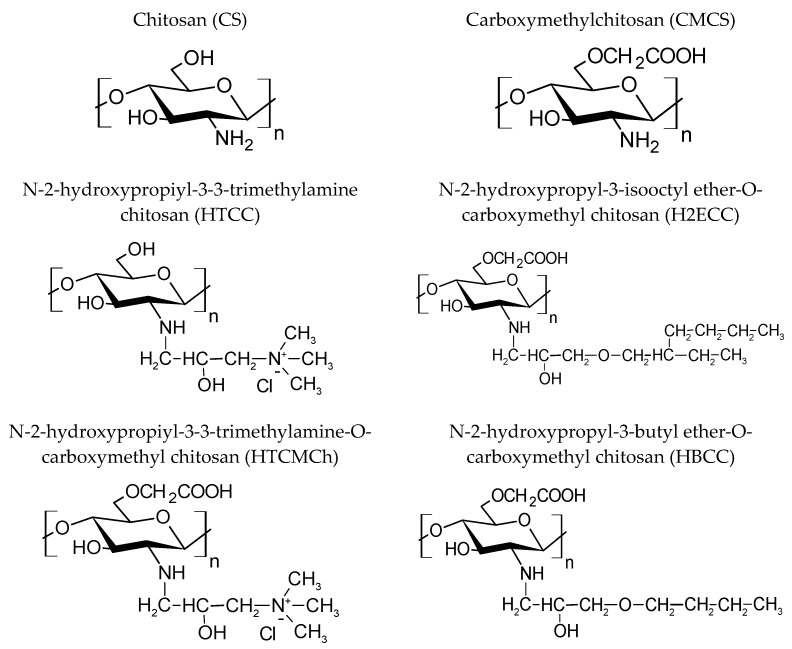
Structural formulas of chitosan derivatives.

The linear polysaccharide chitosan-polyglucosamine (1–4)-2-amino-B-D-glucose [93,94,95] and its synthetic derivatives carboxymethylchitosan and sulfonated chitosan [79,80,129,130] were studied in a methane hydration model and showed high efficiency. In that study [129], four chitosan derivatives were successfully synthesized, and their methane hydrate inhibition effects were compared with those of chitosan (CS) and carboxymethylchitosan (CMCS) (Figure 14, Table 4). Under conditions of 6 MPa, 1 °C, and 400 rpm, the induction time of methane hydrates increased by a factor of 7.3 with the addition of 0.1 wt% (CS). It was found that chitosan with high hydrophobicity can effectively prevent methane gas formation from entering the aqueous solution and reduce the driving force of methane hydrates, resulting in an increase in the induction time. The effect of inhibiting CMCS hydration can be improved by introducing hydroxypropyl-3-trimethylamine and N-2-hydroxypropyl-3-isooctyl ether of the ha groups as a result of the increased molecular hydrophobicity. At the same time, the introduction of a trimethyl quaternary ammonium group increased the ion content in the aqueous solution, which further inhibited the nucleation and growth of methane hydrates.

Varfolomeev M. A. and his colleagues showed that sulfonated chitosan is a promising green oil field reagent that combines the properties of both a kinetic methane hydrate inhibitor and a corrosion inhibitor [80]. Evaluation of hydrate inhibition in a high-pressure autoclave and a micro differential scanning calorimeter showed that hydrate formation was delayed by 14.3 ± 0.2 times, and the amount of hydrate formed decreased by up to 30% compared to water.

Natural biopolymers, as shown by Ankur Singh, show a synergistic effect when applied with commercial KHIs [112]. Three plant polysaccharides, namely, pectin, k-carrageenan, and guar gum, were studied as synergists with four amide kinetic hydrate inhibitors (KHIs), namely, polyvinylpyrrolidone, polyvinylcaprolactam, Luvicap55w, and HIOP. The increase in the effectiveness of hydrate inhibition is characterized by measuring the increase in the delay/induction time (IT) required for hydrate nucleation and the decrease in hydrate growth rate after nucleation. The experimental results obtained using 0.5 wt% of aqueous solutions of the reference KHIs were compared with the results obtained using aqueous solutions prepared by mixing 0.25 wt% of the reference KHIs with 0.25 wt% of the polysaccharide synergists. K-carrageenan showed exceptional inhibitory synergy with all KHIs, with IT increasing by approximately 20–35% with various reference KHIs and the hydrate growth rate decreasing by up to 90%. Guar gum did not increase the IT provided by the reference KHIs. However, it reduced the hydrate growth rate by 77–90%. Pectin demonstrated exceptional synergy with HIOP (commercial KHI) in hydration inhibition, increasing its IT by 45% [112,113].

## 5. Practical Aspects of the Use of Polysaccharides in Inhibiting Hydrate Formation

**Figure 15:**
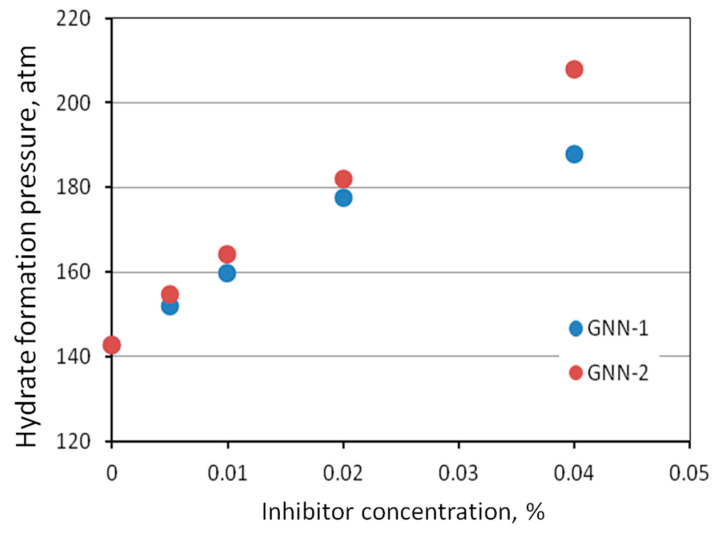
The pressure at the beginning of gas hydrate formation of petroleum gas on the concentration of laboratory formulations of inhibitors.

Based on Na-CMC and arabinogalactan, the authors of [62,63] developed laboratory aqueous formulations of the gas hydrate formation inhibitors GNN-1 and GNN-2. The test of these inhibitors showed an increase in the pressure at the beginning of gas hydrate formation, indicating a more than 70-fold inhibitory effectiveness compared to methanol (Figure 15).

**Figure 16:**
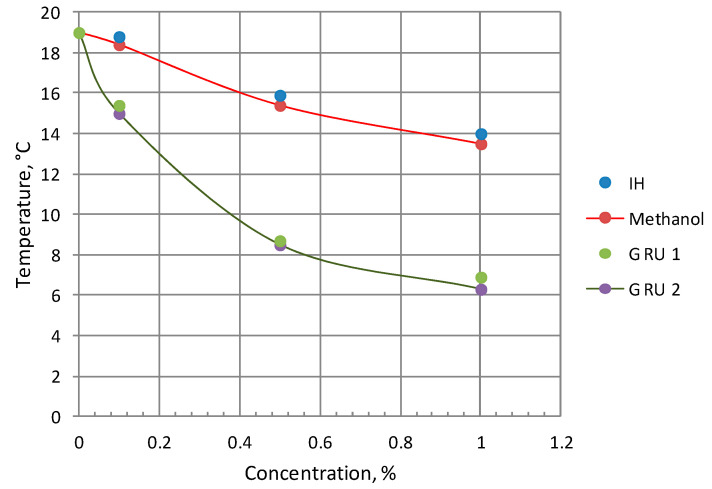
Dependence of the hydrate formation temperature on the inhibitor concentration in an isothermal experiment.

The conducted studies formed the basis for the development of a new synergistic inhibitor of gas hydrate formation (“GRU”), which is composed of alcohols and polysaccharides, to prevent the formation of gas hydrate deposits in gas, gas condensate, and oil and gas wells, as well as in pipeline systems. The comparative assessment of its inhibition effectiveness with the commercial inhibitor of hydrate formation (IH) reagent used in field practice showed that to ensure a given decrease in the temperature of hydrate formation under the conditions of a quasi-isobaric experiment, the required concentration is ~7 times lower (Figure 16).

Through its technological properties, the gas hydrate formation inhibitor “GRU” meets the requirements for commercial gas hydrate inhibitors:

- According to the corrosion aggressiveness of commercial mold, the corrosion rate of carbon steel at 20 °C is 0.0042 g/(m^2^·h);
- The inhibitory effect exceeds the effectiveness of methanol;
- The solidification temperature is −51 °C;
- The kinematic viscosity is 17.7 mm^2^/s at −40 °C;

Pilot field tests were carried out in deposits in Western Siberia with chlorocalcium and sodium bicarbonate reservoir water types. It was found that at a dosage of less than 500 g/m^3^, the formation of hydrate plugs in the annulus of wells was not recorded.

## 6. Conclusions

In this paper, an overview of the current state of use of saccharide polymers in the field of chemical inhibition of gas hydrate formation in the production and transportation of gas-saturated oil is presented. The structure and properties of modern thermodynamic and low-dose (kinetic and anti-agglomerate) gas hydrate formation inhibitors used to prevent and control the formation of gas hydrates in oil are considered. The results of developments made on the basis of a natural polymer, namely, arabinogalactan, and the introduction of a new inhibitor of gas hydrate formation, which is a synergistic combination of thermodynamic and kinetic inhibitors, are presented. The main directions of the development of new types of highly effective and environmentally friendly gas hydrate formation inhibitors based on polysaccharides are analyzed. Promising areas may be:

- functionalization of polysaccharides (introduction of carboxyl, amide and ether fragments);
- increasing the degree of branching of the main chain of polysaccharides;
- search for synergistic additives to polysaccharides and the creation on their basis of new highly effective inhibitors of hydrate formation, economically feasible for industrial use;
- search for the optimal molecular weight of polysaccharides for use as inhibitors of gas hydrate formation.

This work is supposed to serve as an inspiration for the development and modification of green hydrate inhibitors based on polysaccharides in the future.

## Figures and Tables

**Table 1:** Inhibitors of gas hydrate formation.

Type of Hydrate Inhibitors		Name of Chemical Reagents	Note
High-Dosage Hydrate Inhibitors	Thermodynamic Hydrate Inhibitors	Glycols: MEG, TEG. Alcohols: MeOH, EtOH. Salts: NaCl, KCl.	Shift the hydrate-liquid-vapor equilibrium (HLVE) curve; applied in large quantities (10–50 wt%); generally anti-freezing solvents, i.e., methanol, glycols; ineffective in high-sub-cooling conditions.
Low-Dosage Hydrate Inhibitors	Green Hydrate Inhibitors	1. Polysaccharides: chitosan–starch, cellulose ethers. 2. Anti-freeze proteins.	Shift the HLVE curve and retard hydrate formation; applied only at 0.5–2 wt%; generally water-soluble polymers.
	Kinetic Hydrate Inhibitors	Polymers: seven-ring polyvinylcaprolactam, polyvinylpyrrolidone. Ionic liquids.	Delay or retard hydrate formation; applied only at 0.5–2 wt%; generally water-soluble polymers, i.e., PVP, PVCap; ineffective in high-sub-cooling conditions.
	Anti-Agglomerates	Sorbitan: Span20, Span80, Tween.	Do not allow particles to form hydrate plugs; applied only at 0.5–1 wt%; generally surfactants, i.e., Tween and Span series; ineffective in high-water-cut conditions.

**Table 2:** Effective rate and pressure constants for the beginning of gas hydrate formation in the presence of Na-CMC, arabinogalactan, and dextran.

Concentration of Polysaccharide, %	Gas Hydrate Formation Start Pressure, Bar	Effective Rate Constant, *r* × 10^3^, c^−1^	Value of Reduction in the Rate of Gas Hydrate Formation, *k*_MeOH_/*k*_ing_	Effectiveness of Polysaccharide Inhibition α * = *C*_MeOH_/*C*_ing_
Na-CMC				
0	143	4.11	1	1
0.005	168	3.57	1.15	214
0.0065	175	0.91	4.52	277
0.008	185	0.13	31.6	248
Arabinogalactan				
0	143	4.15	1	1
0.005	155	2.11	1.25	170
0.0065	167	0.812	5.11	231
0.008	184	0.193	35.5	263
Dextran				
0	143	4.39	1	1
0.005	169	2.47	1.18	290
0.0065	176	0.633	5.64	255
0.008	183	0.097	45.2	270

* The effectiveness of inhibition *α* = *C*_MeOH_/*C*_ing_ was evaluated пpи Δ*P*_MeOH_ = Δ*P*_ing_ *C*_MeOH_ is the concentration of methanol that provides a pressure reduction of Δ*P*_MeOH_; *C*_ing_—the concentration of the inhibitor that reduces the pressure on Δ*P*_ing_.

**Table 3:** Effect of the molecular weight of Na-CMC on the effectiveness of methane gas hydrate formation inhibition.

Na-CMC	Dosage, %	Temperature of Gas Hydrate Formation, °C	Hydrate Formation Pressure, Bar	Effectiveness, α
Na-CMC-90	0	19	137	1
	0.005	10.6	133	180
	0.010	5.0	131.5	200
	0.050	2.0	125	400
Na-CMC-250	0.005	16.6	133	1
	0.010	13.7	137	40
	0.050	−2.0	131	500
Na-CMC-700	0.005	19	138	-
	0.010	19	136	-
	0.050	19	136	-

**Table 4:** The average induction time of hydrate formation in a methane–water system with 0.1 wt% chitosan at 1 °C, 6 MPa, and 400 rpm.

Additives	P_E_ (MPa)	Average Induction Time (h)	Growth Time (h)
Water	4.14	2.80	9.9
CS	2.98	20.38	15.38
HTCC	2.96	5.90	16.53
CMCS	2.98	2.07	23.67
HTCMCh	3.03	7.70	19.12
HBCC	3.47	3.42	11.17
H2ECC	2.96	3.97	21.52

Additives: hydrate inhibitors; P_E_: reaction equilibrium pressure.

## Data Availability

Not applicable.
